# Epigenome-Wide Scans Identify Differentially Methylated Regions for Age and Age-Related Phenotypes in a Healthy Ageing Population

**DOI:** 10.1371/journal.pgen.1002629

**Published:** 2012-04-19

**Authors:** Jordana T. Bell, Pei-Chien Tsai, Tsun-Po Yang, Ruth Pidsley, James Nisbet, Daniel Glass, Massimo Mangino, Guangju Zhai, Feng Zhang, Ana Valdes, So-Youn Shin, Emma L. Dempster, Robin M. Murray, Elin Grundberg, Asa K. Hedman, Alexandra Nica, Kerrin S. Small, The MuTHER Consortium, Emmanouil T. Dermitzakis, Mark I. McCarthy, Jonathan Mill, Tim D. Spector, Panos Deloukas

**Affiliations:** 1 Wellcome Trust Centre for Human Genetics, University of Oxford, Oxford, United Kingdom; 2 Department of Twin Research and Genetic Epidemiology, King's College London, London, United Kingdom; 3 Wellcome Trust Sanger Institute, Wellcome Trust Genome Campus, Hinxton, United Kingdom; 4 MRC Social, Genetic, and Developmental Psychiatry Centre, Institute of Psychiatry, King's College London, London, United Kingdom; 5 Discipline of Genetics, Faculty of Medicine, Memorial University of Newfoundland, St. John's, Canada; 6 Department of Psychosis Studies, Institute of Psychiatry, King's College London, London, United Kingdom; 7 Department of Genetic Medicine and Development, University of Geneva, Geneva, Switzerland; 8 Oxford Centre for Diabetes, Endocrinology, and Metabolism, University of Oxford, Churchill Hospital, Oxford, United Kingdom; 9 Oxford National Institute for Health Research Biomedical Research Centre, Churchill Hospital, Oxford, United Kingdom; University of Michigan, United States of America

## Abstract

Age-related changes in DNA methylation have been implicated in cellular senescence and longevity, yet the causes and functional consequences of these variants remain unclear. To elucidate the role of age-related epigenetic changes in healthy ageing and potential longevity, we tested for association between whole-blood DNA methylation patterns in 172 female twins aged 32 to 80 with age and age-related phenotypes. Twin-based DNA methylation levels at 26,690 CpG-sites showed evidence for mean genome-wide heritability of 18%, which was supported by the identification of 1,537 CpG-sites with methylation QTLs in *cis* at FDR 5%. We performed genome-wide analyses to discover differentially methylated regions (DMRs) for sixteen age-related phenotypes (ap-DMRs) and chronological age (a-DMRs). Epigenome-wide association scans (EWAS) identified age-related phenotype DMRs (ap-DMRs) associated with LDL (*STAT5A*), lung function (*WT1*), and maternal longevity (*ARL4A*, *TBX20*). In contrast, EWAS for chronological age identified hundreds of predominantly hyper-methylated age DMRs (490 a-DMRs at FDR 5%), of which only one (*TBX20*) was also associated with an age-related phenotype. Therefore, the majority of age-related changes in DNA methylation are not associated with phenotypic measures of healthy ageing in later life. We replicated a large proportion of a-DMRs in a sample of 44 younger adult MZ twins aged 20 to 61, suggesting that a-DMRs may initiate at an earlier age. We next explored potential genetic and environmental mechanisms underlying a-DMRs and ap-DMRs. Genome-wide overlap across *cis*-meQTLs, genotype-phenotype associations, and EWAS ap-DMRs identified CpG-sites that had *cis*-meQTLs with evidence for genotype–phenotype association, where the CpG-site was also an ap-DMR for the same phenotype. Monozygotic twin methylation difference analyses identified one potential environmentally-mediated ap-DMR associated with total cholesterol and LDL (*CSMD1*). Our results suggest that in a small set of genes DNA methylation may be a candidate mechanism of mediating not only environmental, but also genetic effects on age-related phenotypes.

## Author Summary

Epigenetic patterns vary during healthy ageing and development. Age-related DNA methylation changes have been implicated in cellular senescence and longevity, yet the causes and functional consequences of these variants remain unclear. To understand the biological mechanisms involved in potential longevity and rate of healthy ageing, we performed genome-wide association of epigenetic and genetic variation with both chronological age and age-related phenotypes. We identified hundreds of DNA methylation variants significantly associated with age and replicated these in an independent sample of young adult twins. Only a small proportion of these variants were also associated with age-related phenotypes. Therefore, the majority of age-related epigenetic changes do not contribute to rate of healthy ageing at later stages in life. Our results suggest that age-related changes in methylation occur throughout an individual's lifespan and that a proportion of these may be initiated from an early age. Intriguingly, a fraction of the age differentially methylated regions also associated with genetic variants in our sample, suggesting that DNA methylation may be a candidate mechanism of mediating not only environmental but also genetic effects on age-related phenotypes.

## Introduction

DNA methylation is an epigenetic mechanism that plays an important role in gene expression regulation, development, and disease. Increasing evidence points to the distinct contributions of genetic [1], [2], [3], [4], [5], environmental [6], [7], [8], and stochastic factors to DNA methylation levels at individual genomic regions. In addition, DNA methylation patterns at specific CpG-sites can also vary over time within an individual [9], [10] and correspondingly, age-related methylation changes have been identified in multiple tissues and organisms [11], [12], [13], [14], [15]. Although age-related changes in methylation have been implicated in healthy ageing and longevity, the causes and functional consequences of these remain unclear.

Ageing is a complex process, which represents the progression of multiple degenerative processes within an individual. Studies in different organisms have identified many factors that contribute to lifespan and the rate of healthy ageing within an individual. These include components of biological mechanisms involved in cellular senescence, oxidative stress, DNA repair, protein glycation, and others (see [16]). Taking these into account, the concept of biological age has been proposed as a better predictor of lifespan and functional capacity than chronological age alone. Previous studies have proposed that certain traits can be used as measures of biological age [17] and have put forward a stringent definition of an ageing biomarker (see [18]). Here, we examined age-related phenotypes that have previously been considered biomarkers of ageing (see [19]), specifically white cell telomere length, blood pressure, lung function, grip strength, bone mineral density, parental longevity, parental age at reproduction, and serum levels of 5-dehydroepiandrosterone (DHEAS), cholesterol, albumin, and creatinine.

Epigenetic studies of age-related phenotypes can help identify molecular changes that associate with the ageing process. Such changes may include both biological markers of accumulated stochastic damage in the organism, as well as specific susceptibility factors that may play a regulatory role. We explored the hypothesis that epigenetic changes contribute to the rate of ageing and potential longevity in a sample of 172 middle-aged female twins, where methylation profiles and age-DMRs were previously characterized in 93 individuals from the sample [14]. We compared DNA methylation patterns with chronological age in the sample of 172 individuals and related epigenetic variation to age-related phenotypes that have previously been used as biomarkers of ageing. We identified phenotype-associated DNA methylation changes and combined genetic, epigenetic, expression, and phenotype data to help understand the underlying mechanism of association between epigenetic variation, chronological age, and ageing-related traits.

## Results

### DNA methylation patterns in twins associate with genetic variants

**Figure 1:**
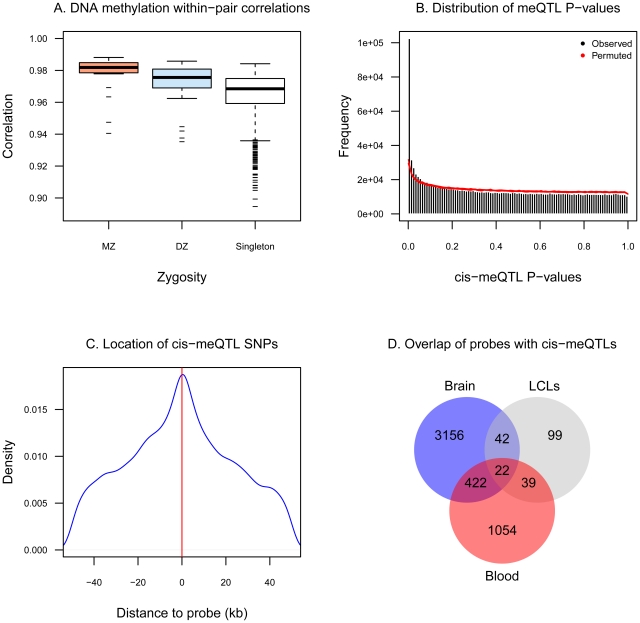
DNA methylation variation associates with genetic variation. A. Genome-wide pair-wise correlation coefficients in 21 pairs of MZ twins, 31 pairs of DZ twins, and 1091 pairs of unrelated individuals. B. Histogram of the observed distribution of P-values (black bars) and the expected distribution (red area indicates 90% confidence region) obtained from ten permutations of the data. C. Majority of SNPs that are *cis*-meQTLs are located within few kb of the methylation probe. D. Overlap of probes that have *cis*-meQTLs from the current study (red) with probes reported to have meQTLs in brain tissues (blue, [3]) and in LCLs (grey, [1]), not accounting for SNP overlap.

We characterized DNA methylation patterns in a sample of 172 female twins at 26,690 promoter CpG-sites that map uniquely across the genome. We observed that the majority of autosomal CpG-sites were un-methylated (beta <0.3, 69% of probes), unlike X-chromosome CpG-sites, which were predominantly hemi-methylated consistent with X-chromosome inactivation (Figure S1). Comparisons of methylation rates within twin pairs indicated that MZ twins had more similar DNA methylation patterns compared to DZ twins, and methylation levels were more similar within co-twins compared to unrelated pairs of individuals (Figure 1A). Correspondingly, intra-class correlation coefficients were significantly greater in MZ twin pairs compared to DZ pairs (Figure S2) indicating evidence for DNA methylation heritability. Estimates of DNA methylation heritability were obtained from CpG-site specific distributions of the MZ and DZ correlation differences. The average whole blood autosomal genome-wide heritability rate was estimated to be 0.182 (genome-wide mean estimate was between 0.176 (95%CI: 0.168–0.185) and 0.188 (95%CI: 0.180–0.196), see Figure S2).

We further investigated methylation heritability by identifying genetic associations with DNA methylation, or methylation QTLs (meQTLs). Methylation QTLs have previously been identified in multiple samples and tissues, and the majority of reported associations have been observed in *cis* and close to the probe [1], [3], [20]. Therefore, we restricted our analyses to *cis*-meQTLs only, that is, SNPs within 100 kb of the methylation probe. At a permutation-based FDR of 5% (P = 1.0×10^−5^), we identified 1,537 probes (6.3% of probes tested) that had cis-meQTLs associations involving 22,849 SNPs (Figure 1B). The majority of associations were obtained for SNPs within a few kb of the methylation probe (Figure 1C). Altogether, of the 1,537 probes with meQTLs identified in this study, 444 (28%) and 61 (34%) were previously reported in brain [3] and lymphoblastoid cell lines [1], respectively (Figure 1D).

Genetic variants that associate with methylation can also have effects on gene expression variation. For the individuals in our sample we also had available gene expression data [21]. We compared the SNPs that were meQTLs in our data with eQTLs from lymphoblastoid cell lines (LCLs) in these individuals, as previously defined [21]. We observed that 10% of previously reported eQTLs in LCLs also had significant meQTL signals in whole blood, suggesting shared mechanisms of methylation and gene-expression regulation in a small proportion of genes, which is consistent with previous findings [1], [3], [5].

### Identification of differentially methylated regions (DMRs) for age and ageing-related phenotypes

**Figure 2:**
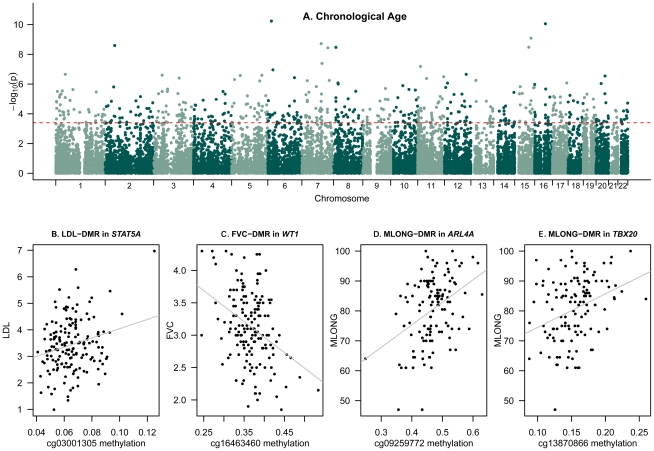
Epigenome-wide association scans of age and age-related phenotypes. (A) Genome-wide results for chronological age at FDR = 5% (a-DMRs). Red dashed line corresponds to FDR 5% significance level threshold. (B–E) Peak ap-DMRs were obtained for (B) LDL-DMR cg03001305, (C) lung function (FVC) DMR cg16463460, and maternal longevity (MLONG) DMRs cg09259772 (D) and cg13870866 (E); grey lines correspond to fitted linear regression models on these data.

We next compared DNA methylation patterns to age and age-related phenotypes by conducting epigenome-wide association scans (EWAS). We fitted a linear mixed effects model regressing methylation levels at each probe on the chronological age of the individuals and included fixed-effect (methylation chip and order of the sample on the chip) and random-effect (family-structure and zygosity) covariates. Differentially methylated regions (DMRs) associated with age (a-DMRs) were identified as those that surpassed the 5% FDR threshold (P = 3.9×10^−4^). We identified 490 a-DMRs in the 172 females twins (Table S1, Figure 2A), of which the majority (98%) exhibited increased methylation with age (hyper-methylated a-DMRs). Of the 490 a-DMRs in our study, 75 hyper-methylated a-DMRs were previously reported as hyper-methylated a-DMRs in a subset of these data (93 individuals from [14]). Furthermore, 36 a-DMRs from our study replicated with the same direction of effect as 88 a-DMRs identified in saliva samples in male twins [11], and 3 a-DMRs were also in the top 10 reported a-DMRs from multiple brain tissues [13]. The a-DMR probes had similar mean levels of methylation, but significantly greater variability (Wilcoxon rank-sum test P<2.2×10^−16^) compared to autosomal CpG-sites across the genome.

The phenotype EWAS DMR analyses focused on the comparison between methylation and age-related phenotypes in the linear mixed effects regression (LMER-DMRs) framework. We examined sixteen phenotypes (Table 1, Figure S3, Table S2), which have previously been studied as biomarkers of age. These phenotypes included telomere length, systolic blood pressure (SBP), diastolic blood pressure (DBP), FEV1 and FVC to examine lung function, grip strength, bone mineral density (BMD), serum levels of DHEAS, serum total cholesterol levels, serum high density cholesterol levels (HDL), calculated levels of serum low density cholesterol (LDL), serum albumin levels, serum creatinine levels, maternal longevity (MLONG), paternal longevity (PLONG), maternal age at reproduction (MREPROD), and paternal age at reproduction (PREPROD). For each phenotype we regressed methylation levels against the phenotype and included methylation chip and order on the chip as fixed-effect covariates and family and zygosity as random effects. We also performed the analyses by including or excluding chronological age as a fixed effect covariate. We examined the results using a permutation-based significance threshold, by preserving twin-structure and taking into account missing data patterns for each phenotype and evidence of co-methylation and deviations from normality in the DNA methylation data. We observed that four ap-DMRs for LDL (cg03001305 in *STAT5A* with LDL: age-corrected methylation∼LDL beta = 4.73×10^−3^, se = 8.75×10^−4^, P = 8.72×10^−7^), lung function (cg16463460 in *WT1* with FEV1: methylation∼FEV1 beta = −0.035, se = 6.72×10^−3^, P = 5.31×10^−7^; cg16463460 in *WT1* with FVC: methylation∼FVC beta = −0.0293, se = 5.59×10^−3^, P = 4.67×10^−7^), and maternal longevity (cg09259772 in *ARL4A* with MLONG: methylation∼MLONG beta = 2.11×10^−3^, se = 4.21×10^−4^, P = 1.83×10^−6^; cg13870866 in *TBX20*: methylation∼MLONG beta = 1.10×10^−3^, se = 2.11×10^−4^, P = 1.21×10^−6^) were genome-wide significant at a permutation-based FDR of 5% (Figure 2, Figure S4). We repeated the LMER-DMR analyses using normalized methylation levels and observed that the reported FDR 5% ap-DMRs (Table 1) also fell in the top-ranked results from the normalized methylation DMR analyses.

**Table 1:** Age and age-related phenotype EWAS DMR results.

Phenotype^a^	Data (%)	Age Effect^b^	EWAS LMER-DMRs^c^	EWAS MZ-DMRs^e^
Age	100	*NA*	490 age DMRs	*NA*
Telomere length	62.2	−0.030±0.009	-	*NA*
SBP	100	0.663±0.146	-	-
DBP	100	−0.019±0.098	-	-
Lung function	97.7	−0.028±0.006	cg16463460 (*WT1*)	-
Grip strength	64.0	−0.451±0.081	-	*NA*
BMD	86.7	−0.005±0.001	-	-
Serum DHEAS	99.4	−0.023±0.007	-	-
Cholesterol	97.1	0.052±0.012	-	cg01136458 (*CSMD1*)
HDL	97.1	0.016±0.012	-	-
LDL	94.8	0.018±0.011	cg03001305 *(STAT5A)* d	cg01136458 (*CSMD1*)
Serum Albumin	91.9	−0.102±0.030	-	-
Serum Creatinine	86.0	0.120±0.102	-	-
MLONG	73.8	2.3×10^−6^±2.8×10^−4^	cg09259772 (*ARL4A*)cg13870866 (*TBX20*)	*NA*
PLONG	73.3	4.1×10^−6^±1.7×10^−4^	-	*NA*
MREPROD	80.8	6.3×10^−6^±5.3×10^−4^	-	*NA*
PREPROD	82.0	6.2×10^−6^±4.5×10^−4^	-	*NA*

a Phenotypes are listed as follows: Telomere length, systolic blood pressure (SBP), diastolic blood pressure (DBP), Lung function (FVC), grip strength, bone mineral density (BMD), serum levels of DHEAS, serum total cholesterol, high density cholesterol (HDL), low density cholesterol (LDL), serum albumin, serum creatinine, maternal longevity (MLONG), paternal longevity (PLONG), and maternal age at reproduction (MREPROD), and paternal age at reproduction (PREPROD).

b Regression coefficient estimate from the linear mixed effect regression model regressing raw phenotype on chronological age (age regression coefficient +/− se).

c LMER-DMR results are shown at a permutation-based FDR threshold of 5%.

d Results were significant when age was included as a fixed-effect covariate.

e MZ-DMRs are shown at FDR 5% threshold, including age correction.

We compared the 490 a-DMRs to ap-DMRs. Only one of the 490 a-DMRs was also significantly associated with ageing-related phenotypes, specifically ap-DMR for maternal longevity (*TBX20*). We examined the genome-wide distribution of ap-DMR association *P*-values in the set of a-DMRs, but did not observe an enrichment of ap-DMRs in the set of a-DMRs compared to random sets of probes (Figure S5).

We tested for correlation in DNA methylation (co-methylation) between nearby CpG-sites both genome-wide and specifically at the 490 a-DMR CpG-sites. We observed evidence for co-methylation, that is, pairs of CpG-sites located within 1–2 kb apart showed greater correlation in methylation patterns compared to pairs of CpG-sites located further apart. The pattern of co-methylation was also observed at the a-DMR CpG-sites, in particular DNA methylation levels at CpG-sites located within 500 bp of an a-DMR were highly correlated with the a-DMR DNA methylation levels compared to CpG-sites located further away from a-DMRs (Figure S6).

To assess if the DMRs identified in our study capture differential proportion of whole blood cell (WBC) sub-types we compared DNA methylation levels with WBC sub-type proportions for neutrophils, eosinophils, monocytes, and lymphocytes. Blood count DMR analyses were performed at the 493 a-DMRs and ap-DMRs, and results are presented at a DMR Bonferroni corrected *P*-value = 0.05 (nominal P = 1×10^−4^). We did not observe significant associations between DNA methylation at the 490 a-DMR probes with proportion of neutrophils, eosinophils, or monocytes in our data. However, at 19 a-DMRs (3.9% of a-DMRs) DNA methylation levels were significantly associated with lymphocyte counts (Table S3), suggesting that the a-DMR effects at these probes may in part reflect variability in the number of lymphocytes over time. We did not observe significant associations between DNA methylation levels at the four ap-DMRs with any of the blood cell sub-types tested. We conclude that variability in WBC sub-types does not have a major effect on age and age-related DMRs in our study.

### Genetic associations for age-related traits may be mediated by DNA methylation

**Figure 3:**
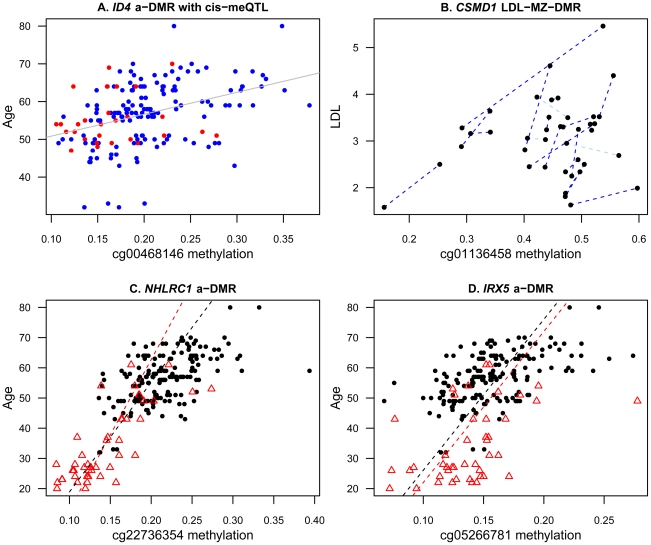
Examples of age and age-related phenotype DMRs in the discovery and replication samples. (A) Example of an a-DMR probe (cg00468146 in *ID4*), which also has *cis*-meQTLs. Individuals are coloured according to *cis*-meQTL rs12660828 genotype (AG = red, GG = blue). (B) MZ twin methylation difference analyses identify potential environmentally-triggered DMR cg01136458 in *CSMD1* associated with LDL. MZ co-twins are linked by dark blue (positive DMR effect) or light blue (negative DMR effect) dashed lines. (C,D) The two most associated a-DMRs (in *NHLRC1* (C) and *IRX5* (D)) in the discovery sample of 172 individuals (black dots) also replicate in the sample of 44 younger individuals (red triangles). Dashed lines represent estimated effects within the discovery (black) and replication (red) sample.

To explore potential mechanisms underlying a-DMRs and ap-DMRs in our sample, we first considered the hypothesis that DMR effects may mediate genetic-phenotype associations. We focused specifically on the overall set of 493 identified DMRs for age (490 a-DMRs) and age-related phenotypes (4 ap-DMRs). We observed that 5% of these DMRs also had *cis*-meQTL effects, which was lower than the genome-wide rate of 6.3% of probes on the array with *cis*-meQTLs. Altogether, the DMRs with *cis*-meQTLs were located in 26 genes and some of the genes had previously reported genetic associations with longevity (a-DMR *MEFV* [22]) or had been implicated in longevity and ageing (a-DMRs *SMPD3* [23], *GALR1* [24], [25], *ID4* [26]; see Figure 3A). Therefore, genetic and methylation effects may impact age-related phenotypes in a small proportion of genes, either with independent effects or by mediating genetic-phenotype associations through DNA methylation.

To explore this hypothesis further on a genome-wide level, we estimated the extent to which *cis*-meQTLs, genotype-phenotype associations, and ap-DMRs overlapped in our data. We performed genome-wide association scans (GWAS) for 12 phenotypes in the set of 172 twins. We assessed the overlap between: (1) SNPs that were *cis*-meQTLs and were also phenotype-GWAS-QTLs, (2) phenotypes with GWAS-QTLs that also had ap-DMRs, and (3) CpG-sites with meQTLs that were also ap-DMRs. We compared the overlap in the observed data to two genome-wide permutations of the analyses.

There were 1,537 CpG-sites associated with 22,849 *cis*-meQTLs SNPs in our data. Of the 22,849 SNPs, 344 SNPs (which were originally *cis*-meQTLs for 111 CpG-sites) also showed modest suggestive evidence for association in the phenotype-GWAS analyses for each trait (at P = 0.001). Of the 111 CpG sites, 16 CpG-sites (with 53 SNPs) also had suggestive evidence for ap-DMR signals (P = 0.01), where the CpG-site was associated with the same phenotype as the GWAS QTL SNPs (which were also cis-meQTLs for that CpG-site). Altogether, we observed 1% (16 of 1,537 probes) three-way overlap across the analyses combining the 12 phenotypes, and up to 0.2% overlap for individual phenotypes (for BMD, Cholesterol, DBP, DHEAS, FVC, HDL, and Telomere length; see Table S4). In all cases, a SNP genotype was associated with both CpG-site methylation and phenotype, and the CpG-site methylation was also associated with the phenotype, suggesting that these are likely genotype-phenotype associations that may be mediated through DNA methylation. We estimated the expected overlap of results under the null hypothesis that methylation does not mediate genotype-phenotype associations by permuting the methylation data only, preserving twin structure and patterns of co-methylation, for two genome-wide permutations. We selected the top 1,537 CpG-sites that showed most associations with *cis*-meQTL SNPs in the permutations, and assessed the proportion of CpG-sites that showed suggestive methylation-phenotype associations (P = 0.01) and had *cis*-meQTLs SNPs that showed suggestive genotype-phenotype associations (P = 0.001). In both replicates, we observed minimal overlap of probes across the three sets of the analyses under the null hypothesis (mean overlap 0.36% or 5.5 probes of 1,537 overlapped under the null).

### Age-related DNA methylation differences in monozygotic twins

Epigenetic variants may also accumulate independent of the genetic sequence, because different lifestyle choices and environments may trigger epigenetic changes. The recently reported association between smoking and methylation levels in *F2RL3* is likely to be an example of such effects [6]. Therefore, we next tested for ageing-phenotype associated methylation variants that appeared uncorrelated with genetic variation, by comparing methylation and phenotype differences within monozygotic twin pairs (MZ-DMRs). We limited analyses to 21 MZ twin pairs and 12 phenotypes for which at least 12 of the 21 pairs had phenotype data available for both twins (Table 1). At a permutation-based FDR of 5%, we observed one MZ-DMR (cg01136458, P = 3.12×10^−7^) in the promoter of the CUB and Sushi multiple domains 1 gene (*CSMD1*) that associated with total cholesterol and LDL (Figure 3B). Genetic variants in *CSMD1* have previously been associated with several complex traits in multiple studies, but we did not observe an enrichment of ap-DMR or MZ-DMR signals in this gene for the other age-related phenotypes in our data.

### Replication of age DMRs in younger adult twins

We pursued replication of the 490 a-DMRs in a sample of 44 younger adult MZ twins (age range 20–61, median age 28), who were discordant for psychosis [27]. In the overall set of 44 twins, we replicated 184 a-DMRs (38%) with the same direction of effect at a nominally significant threshold (P = 0.05). In the set of 22 unaffected unrelated individuals alone, 69 a-DMRs (14%) replicated with the same direction of association at nominal significance. Given the relatively modest sample size, we also examined the direction of the association between methylation and age without considering significance thresholds. We observed that 404 a-DMRs (82%) showed consistent effects in the overall set of 44 twins, and 369 a-DMRs (77%) had consistent effects in the set 22 unaffected unrelated individuals alone. The two most significant a-DMRs (cg22736354 in *NHLRC1* and cg05266781 in *IRX5*) showed consistent effects in both discovery and replication samples (Figure 3C, 3D). Both a-DMRs were hyper-methylated with age in the discovery (cg22736354 methylation∼age beta = 2.76×10^−3^, se = 3.73×10^−4^; cg05266781 methylation∼age beta = 2.00×10^−3^, se = 3.03×10^−4^) and replication (cg22736354 methylation∼age beta = 2.01×10^−3^, se = 3.03×10^−4^; cg05266781 methylation∼age beta = 2.00×10^−3^, se = 4.87×10^−4^) samples.

### Functional characterization of age DMRs

We explored the functional role of a-DMRs by studying their genome localization, by comparing the a-DMR methylation data to gene expression estimates from LCLs, and by searching for gene ontology terms associated with the a-DMR genes.

**Figure 4:**
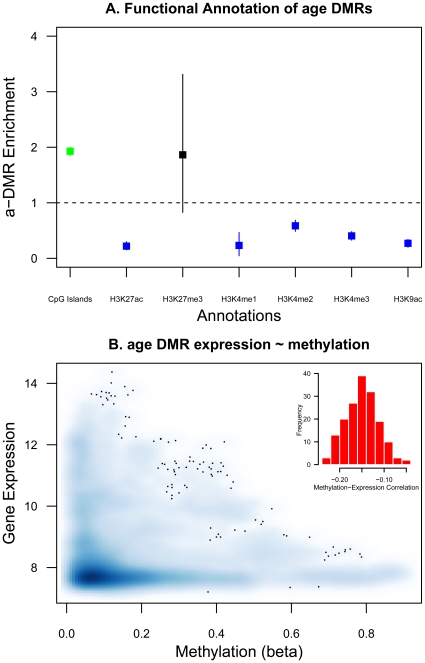
Functional characterization of a-DMRs. A. Enrichment and depletion of a-DMRs in functional genomic categories. Enrichment is calculated as the proportion of a-DMRs in each functional category (CpG islands (green) or HapMap CEPH LCL histone peaks (blue, black)) over the proportion of 26,690 probe in that functional category. Bars represent the 95% bootstrap percentile confidence intervals. B. Whole blood methylation and LCL gene expression estimates in the age DMR genes show significant negative correlation (histogram shows the distribution of gene-based rank correlation coefficients between methylation and gene expression).

We first characterized the a-DMRs by examining their location with respect to functional genomic annotations and other epigenetic signature marks. We considered functional categories with respect to CpG islands, histone modification marks in LCLs, and DNA binding motifs. For each category we assessed the enrichment or depletion of a-DMR probes relative to all 26,690 probes (Figure 4A). We found an enrichment of a-DMRs in CpG islands (see Figure 4A), which is consistent with previous observations for hyper-methylated a-DMRs [14], [28]. We also observed a depletion of a-DMRs in the presence of histone marks that target active genes in LCLs (Figure 4A). For example, a-DMRs were under-represented in H3K27ac, H3K4me3, and H3K9ac peaks, which are indicative of enhancers or transcriptional activity, and have been positively correlated with transcription levels.

To search for an enrichment of DNA binding motifs in the set of 435 a-DMR genes, we used PSCAN [29] with the JASPAR database [30]. We found a significant enrichment for 28 transcription factor binding sites, many of which could play a role in ageing (Table S5). The transcription factors associated with enriched DNA binding sites were involved in development (PLAG1), cellular senescence (Mycn), regulation of cell cycle (Egr1, CTCF, E2F1), or had also been associated with ageing (NF-kappaB), age-related processes (NFKB1, Klf4, MIZF, Mafb, ESR1) or other established ageing-related genes such as *WRN* (SP1, TFAP2A, Myc, Mycn), *TERT* (Myc), and *TORC1* (HIF1A::ARNT).

To explore the functional consequences of a-DMRs, we examined gene-expression data at the a-DMR genes, using gene expression estimates obtained in LCLs from the same individuals [21]. We compared whole blood DNA methylation to LCL gene expression in 168 individuals at 348 genes, which had methylation CpG-sites within 2 kb of the transcription start site. We found significant negative correlations between methylation and gene expression in the set of a-DMR genes (Figure 4B), and an overall trend towards low levels of expression at a-DMR genes. One caveat applying to this analysis is that blood methylation corresponds to multiple cell types including lymphocytes.

We performed gene ontology term enrichment analyses of biological processes and molecular functions in the set of 435 a-DMR genes [31]. The results indicated strong enrichment for genes involved in the regulation of developmental morphological processes, DNA binding, regulation of cell differentiation, regulation of transcription, and regulation of metabolic and biosynthetic processes (Table S6).

## Discussion

We identify hundreds of CpG-sites that exhibit age-related directional changes in methylation. The majority of these effects are hyper-methylated with age, a large proportion replicate in an independent sample, and some changes are observed in multiple tissues. These findings indicate that a-DMRs are not likely stochastic events, but instead may associate with biological mechanisms involved in ageing and potential longevity. To address this we compared methylation variants to measures of biological ageing, focusing on markers like telomere length and other age-associated phenotypes that have previously been linked to ageing. However, our phenotype-methylation comparisons identified only a small subset of a-DMRs that also associate with ageing related traits. These findings suggest that although a-DMRs do not appear to be random events, the majority of observed a-DMRs may either be neutral (or of very small individual effect) to measures of biological age at later stages in life, or may relate to as yet unknown pathways that correlate with biological ageing.

The a-DMRs we detected in blood overlap with previously reported a-DMRs obtained in saliva and brain samples, and previous observations also show that some hyper-methylated a-DMRs occur in both blood and buccal tissues [14]. These results indicate that a proportion of a-DMRs are conserved across tissues in samples of different ages and genders, and raise the question of when such age related methylation changes occur during an individual's lifespan and what their functional role is. Functional annotation of a-DMRs show an enrichment of genes involved in regulation of development, morphology, regulation of transcription, and DNA binding, which has also been previously observed in brain samples [13]. The genes nearest to a-DMRs also showed an enrichment of DNA binding motifs for transcription factors linked to ageing. Functional genomic annotation indicated that a-DMRs tend to associate with epigenetic marks targeting low levels of transcription. Consistent with this, a-DMR genes showed predominantly low levels of expression in LCLs and significant negative correlations between methylation and gene expression. Altogether, we find that a-DMRs are located in regions of the genome that functionally link to development and ageing, and tend to show low gene expression rates in our sample of middle-aged individuals.

DNA methylation plays a key role in development and tissue differentiation and therefore, it is plausible that at some a-DMRs differential methylation patterns are established early on in development prior to tissue differentiation and continue to intensify over time. For example, CpG-sites that are methylated during early development may become hyper-methylated over time, either because such sites are more prone to methylation or because cells carrying the methylated variant are more likely to replicate. Our findings indicate that age-related changes in methylation occur throughout life, but the timing of the initial age-related trigger at each CpG-site remains unclear. Our results are consistent with a model where at some CpG-sites the initial change may occur during development and early life, but specifically at an age prior to adulthood. Age DMR studies of younger samples could be useful in establishing the proportion of a-DMRs that are also observed at earlier stages in life. We were able to replicate up to 38% of a-DMRs in a sample of younger adults, but samples from newborns or samples obtained prior to tissue differentiation would help resolve the question of when a-DMRs are established, especially tissue conserved a-DMRs.

We tested for methylation associations with age-related phenotypes (ap-DMRs) to gain insight into potential mechanisms underlying a-DMRs. We identified four ap-DMRs, of which only one (cg13870866 in *TBX20*) was also an a-DMR. Two of the ap-DMRs were in genes already implicated in ageing, longevity, or cell senescence, *STAT5A* [32] and *WT1* [33]. Our genotype-methylation-phenotype overlap results suggest that in a small proportion of genes DNA methylation may be a candidate mechanism of mediating genetic association effects on ageing-related phenotypes, however, we cannot exclude the possibility that rare genetic variants in the methylation probe sequence drive some of these associations. We also assayed DNA methylation levels at the four ap-DMR probes in 48 of the individuals in the current study using the new Illumina Infinium HumanMethylation450 BeadChip and obtained significant positive correlations in DNA methylation levels at three ap-DMRs (cg16463460, cg09259772, and cg13870866), indicating evidence for technical validation at these probes.

A difficult question in epigenetic studies of phenotypic data is establishing the timing of the epigenetic change relative to trait progression. The age-related phenotype methylation changes identified here may occur prior to the phenotypic change and potentially contribute to phenotype variation, or they may occur as a consequence of ageing processes in the cell. In this cross-sectional study we cannot establish the timing of ap-DMRs with respect to phenotype progression, but can use the findings as potential markers of rate of ageing.

Regions that exhibit evidence for DNA methylation heritability, such as the *IGF2/H19* region, also exhibit more stable DNA methylation levels over time and tend to occur in functionally important promoter regions [4]. Epigenetic variants in heritable methylation regions are likely to be present at birth, to be more stable over time, and may be involved in regulating the rate of ageing. In our study, 26 a-DMRs also had *cis*-meQTLs and represent a candidate set of heritable DNA methylation regions that are likely to be more stable and may be involved in longevity. On the other hand, environment-dependent changes in DNA methylation in MZ twins have been reported to occur preferentially in gene-poor regions (see [34]). Here, we identify *CSMD1* as the most likely example of an environmentally driven DMR for LDL, but this gene does not fall in a gene-poor locus.

The methylation heritability estimates obtained in our data, 0.176 and 0.188 (Figure S2), are slightly greater than those previously reported for whole-blood methylation [4], which may be due to the difference in regions assayed by the two arrays and to the promoter locations of our probes. Correspondingly we identified 1,537 CpG-sites with meQTLs. It is possible that a proportion of the meQTLs in our data are due to linkage disequilibrium between the cis-meQTL SNPs and unknown genetic variants in the probe sequence. Obtaining genetic sequences for these individuals will establish the extent to which rare-probe variants exist and affect meQTL findings. However, the overlap across probes with meQTLs across studies and tissues suggests that a significant proportion of the QTLs are conserved across tissues [35], [36]. These are likely to exhibit stable patterns of methylation across mitosis and meiosis, and may be of functional importance.

Many factors will impact the power to detect differential methylation effects related to age and age-related phenotypes. One of these factors relates to the coverage and precision of the methylation assay. In our case, the coverage of methylation sites on the Illumina27k array is relatively sparse and promoter-specific, and therefore limits power to detect age related methylation changes. It is likely that additional age related changes in methylation may be identified using higher resolution methylation assays in larger sample sizes of wider age ranges.

In this study, we identified methylation changes associated with chronological age and ageing-related phenotypes and we explored mechanisms underlying ageing-related changes in DNA methylation. Both environmental and genetic factors are thought to contribute to healthy ageing, and epigenetic mechanisms represent a potential pathway of mediating these effects on ageing and age related traits.

## Materials and Methods

### Ethics statement

All samples and information were collected with written and signed informed consent. The study was approved by the local research ethics committee.

### Phenotype data

Phenotype data were obtained for 172 female twins from the TwinsUK cohort. The TwinsUK cohort (St Thomas' UK Adult Twin Registry) comprises unselected volunteers ascertained from the general population [37]. Means and ranges of quantitative phenotypes in Twins UK were similar to age-matched samples from the general population in the UK [38]. The 172 twins in this study included 33 MZ pairs, 43 DZ pairs, and 20 singletons. Phenotypes used in the current study included telomere length, systolic blood pressure (SBP), diastolic blood pressure (DBP), forced expiratory volume in one second (FEV1) and forced expiratory vital capacity (FVC) to examine lung function, grip strength, bone mineral density (BMD), serum levels of DHEAS (DHEAS), serum total cholesterol levels, serum high density cholesterol levels (HDL), calculated levels of serum low density cholesterol (LDL), serum albumin levels (Albumin), serum creatinine levels (Creatinine), maternal longevity (MLONG), paternal longevity (PLONG), maternal age at reproduction (MREPROD), and paternal age at reproduction (PREPROD). Phenotype data used in the current study were previously described in the Twins UK sample for the majority of phenotypes, specifically for telomere length [39], [40], blood pressure [41], lung function [42], grip strength [43], BMD [43], [44], [45], DHEAS [46], serum cholesterol [47], [48], serum albumin [49] and serum creatinine [50]. Parental longevity data were obtained by questionnaire in 2011, and included parental age at death and parental age at reproduction for each individual. In cases of missing data, we used co-twin estimates to infer values. In rare cases parental age at death estimates varied across co-twins, and if the estimates were within one year we took the mean, otherwise data were assigned as missing. In 171 of the individuals from our sample we also obtained white blood cell (WBC) sub-type counts [51]. WBC counts were derived from fluorescence activated cell sorting of peripheral blood. WBC sub-type specific cell counts were calculated by multiplying the proportion of the WBC count comprised by each cell type by the total WBC cell count (estimated in thousands of cells per ml), for four cell types in our sample: neutrophils, eosinophils, monocytes, and lymphocytes.

### Illumina Methylation27K data

DNA methylation levels were obtained in 172 middle-aged (age range 32–80, median age 57) healthy female volunteers who were twins, including monozygotic (MZ) twins, dizygotic (DZ) twins, and unrelated individuals. DNA methylation patterns were assayed in two batches of 93 [14] and 79 samples. We considered 26,690 probes that mapped uniquely to the human genome (hg18) within 2 mismatches (see [1]) and discarded probes with missing data, resulting in a final set of 24,641 autosomal probes and 959 X-chromosome probes. Methylation values are reported as betas, which represent the ratio of array intensity signal obtained from the methylated beads over the sum of methylated and unmethylated bead signals. We performed principal components analysis (PCA) of the methylation values (normalized to N(0,1) at each probe) and correlated the first five principal component (PC) loadings to covariates (age, methylation arrays, order of the sample on the methylation array) to identify potential confounders. We observed that both methylation array and order of the sample on the array were significantly correlated with the first and second PCs and therefore included these two variables as fixed-effect covariates in the linear mixed effects models used in the majority of downstream analyses. Further analyses of DNA methylation patterns within twins were performed using intraclass correlation coefficients (ICC) using the R package irr (v0.82). Twin-based DNA methylation heritabilities were estimated as 2(ICC_MZ - ICC_DZ), and were calculated within each batch of data separately.

### Genotype data

Direct genotypes were available for 171 samples on a combination of Illumina platforms (HumanHap300, HumanHap610Q, 1M-Duo and 1.2MDuo 1M custom arrays) and stringent quality control checks were applied to these genotype data as previously described [21], [52]. HapMap genotypes were imputed in the set of 171 individuals. Imputation was performed in Impute (v2 [53]) using two reference panels, P0 (HapMap2, rel 22, combined CEU, YRI and, ASN panels) and P1 (610K+, including the combined HumanHap610K and 1M array). After imputation, SNPs were filtered at a MAF>5% and an Impute info value of >0.8. Altogether, there were 2,054,344 directly genotyped and imputed autosomal SNPs used in the QTL analyses.

### Gene expression data

Gene expression estimates and eQTLs from lymphoblastoid cell lines (LCLs) in the samples were obtained for 168 individuals in the study [21]. Gene expression levels were measured using the Illumina expression array HumanHT-12 version 3 as previously described [21]. Each sample had three technical replicates and log2 - transformed expression signals were quantile normalized first across the 3 replicates of each individual, followed by quantile normalization across all individuals [21]. We assigned methylation and expression probes to the gene with the nearest transcription start site using Refseq gene annotations. For each gene we obtained the mean methylation (or gene expression) estimate, by averaging values over multiple methylation (or gene expression) probes if more than one probe was assigned to that gene. There were altogether 435 genes nearest to the 490 age DMRs, of which 348 had transcription start sites within 2 kb of the methylation CpG-sites and for which we also had whole blood methylation data and LCLs gene expression data in 168 individuals. Linear mixed effects models and Spearman rank correlations were used to compare methylation and expression data per gene.

### Methylation QTL analyses

We tested for methylation QTLs at 24,522 autosomal probes, which had at least one SNP within 50 kb of the probe that passed genotype QC criteria. We fitted a linear mixed-effects model, regressing the methylation levels at each probe on fixed-effect terms including genotype, methylation chip, and sample order on the methylation chip, and random-effect terms denoting family structure and zygosity. Prior to these analyses, the methylation values at each CpG-site were normalized to N(0,1). Results from meQTL analyses are presented at a false discovery rate (FDR) of 5%, estimated by permutation. Here, we permuted the methylation data at the 24,522 autosomal probes, performed *cis* association analyses on the permuted and normalized methylation data, and repeated this procedure for 10 replicates selecting the most associated SNP per probe per replicate. FDR was calculated as the fraction of significant hits in the permuted data compared to the observed data at each p-value threshold.

### DMR analyses

Linear mixed effects models were used to assess evidence for DMRs. In the a-DMR analyses we regressed the raw methylation levels at each probe on fixed-effect terms including age, methylation chip, and sample order on the methylation chip, and random-effect terms denoting family structure and zygosity. To assess the significance of the a-DMRs we compared this model to a null model, which excluded age from the fixed-effects terms. In the ap-DMR analyses we regressed the raw methylation levels at each probe on fixed-effect terms including phenotype, methylation chip, and sample order on chip, and random-effect for family and zygosity, and compared the fit of this model to a null model which excluded the phenotype. We also performed the ap-DMR analyses by including age as a fixed effect covariate in both the null and alternative models. We also repeated both the a-DMR and ap-DMR analyses using normalized methylation levels (to N(0,1)) and observed that the reported DMRs were top-ranked in the normalized analyses. To assess genome-wide significance we performed 100 permutations and estimated FDR by calculating the fraction of significant hits in the permuted data compared to the observed data at a specific P-value threshold.

Monozygotic twin DMR effects were calculated in the set of 21 MZ twin pairs where both twins were assayed within the same batch of methylation arrays. We estimated MZ-DMRs for 12 phenotypes where data were available in at least 12 MZ pairs. For each phenotype of interest we fitted a linear model comparing phenotype within-pair differences to methylation within-pair differences and reported the P-values obtained from the F-statistics from the overall regression. For the age-corrected analyses we fitted the regression including age as a covariate and compared the results to a null model, which included phenotype differences and age alone. We performed 100 replicates to estimate FDR 5% significant results as described above. At the FDR 5% significance threshold (nominal P = 2.03×10^−6^), we estimated 35% power to detect the observed correlation (Pearson correlation = 0.83) between methylation MZ-differences at cg01136458 in *CSMD1* (mean MZ-beta-difference = 5%) and LDL MZ-differences (mean MZ-LDL-difference = 0.73 SD) in 20 MZ pairs.

### Age DMR replication

The replication sample comprised 44 MZ twins discordant for psychosis, that were profiled on the Illumina 27K array as previously described [27]. The sample consisted of younger adults (age range 20–61, median age 28), including both female and male twin pairs. We compared methylation against age at the 490 a-DMRs both in the entire set of 44 twins and in the set of 22 unaffected unrelated individuals. In the set of 44 twins we fitted linear mixed effect models, regressing the normalized beta values per probe (normalized to N(0,1)) against methylation chip, sample order on the chip, sex, and age as fixed effects, and family as random effect. In the set of 22 unaffected unrelated individuals comprising the control twin from each pair we calculated Spearman rank correlation coefficients on the untransformed methylation beta values against age.

### Genome-wide association scans

Genome-wide association scans were performed using linear mixed effects models for 12 phenotypes including telomere length, systolic blood pressure (SBP), diastolic blood pressure (DBP), FEV1 and FVC to examine lung function, grip strength, bone mineral density (BMD), serum levels of DHEAS, serum total cholesterol levels, serum high density cholesterol levels (HDL), calculated levels of serum low density cholesterol (LDL), serum albumin levels, and serum creatinine levels. Linear models were fit as described in the meQTL analyses section substituting phenotype for methylation, using an additive model. SNPs with evidence for association that surpassed P = 0.001, were considered in the overlap across *cis*-meQTL, genotype-phenotype, and DMR findings.

### Functional characterization of DMRs

The 26,690 methylation probes were assigned to CpG islands according to previous definitions [54], resulting in 11,299 CpG sites that were in CpG islands and 15,391 that were outside of CpG islands. Histone modification ChIP-seq data were obtained from the Encode project from one CEPH HapMap LCL (GM12878) in the UCSC genome browser. Peaks in the genome-wide read-depth distribution from ChIP-seq histone modifications H3K9ac, H3K27ac, H3K27me3, H3K4me1, H3K4me2, and H3K4me3 were obtained as previously described (see [1]). Enrichment a-DMR estimates were calculated as the proportion of a-DMRs in each functional category (CpG islands or histone peaks) over the proportion of 26,690 probe in that functional category. Enrichment 95% confidence intervals were estimated using bootstrap percentile intervals of 1,000 re-samplings of the a-DMR data per annotation category.

Gene ontology term enrichment analysis was performed using the GOrilla tool for identifying enriched GO terms in the ranked list of a-DMR genes [31], using Refseq gene annotations in the entire set of 26,690 probes as background.

## Supporting Information

Figure S1 Summary characteristics of DNA methylation patterns in 172 female twins. Distribution of methylation scores (beta) in (A) autosomal and (B) X-chromosomal probes in all individuals.
(PDF)

Figure S2 Distribution of intra-class correlation coefficients (ICC) in twins. Density plots of ICC in MZ twins (red) and DZ twins (blue) for two batches of methylation data (batch 1 consists of 93 twins (left) and batch 2 consists of 79 twins (right)). The mean MZ-ICCs and DZ-ICCs were estimated as 0.257 and 0.168 in batch 1 (MZ-ICC *vs* DZ-ICC P<2×10^−16^), and as 0.3557 and 0.261 in batch 2 (MZ-ICC *vs* DZ-ICC P<2×10^−16^). The corresponding methylation probe heritabilities were calculated as 2(ICC_MZ - ICC_DZ) and the genome-wide estimates were 0.176 (95%CI:0.168–0.185) and 0.188 (95%CI:0.180–0.196) for the data in batch 1 (left) and batch 2 (right), respectively.
(PDF)

Figure S3 Correlation across age-related phenotypes. Below diagonal plots represent each pair of phenotypes and the corresponding rank correlation coefficient is shown above the diagonal.
(PDF)

Figure S4 EWAS results for age-related phenotypes. FDR 5% ap-DMRs were obtained for (A) LDL, (B) lung function (FVC), and (C) maternal longevity (MLONG) with (green) and without (blue) age-correction. Red dashed lines correspond to age-corrected (A) and non-age-corrected (B,C) analysis FDR 5% levels.
(PDF)

Figure S5 Lack of enrichment of age-related phenotype DMR association in the set of age DMRs.
(PDF)

Figure S6 Evidence for co-methylation. Spearman correlation in methylation levels between all pair-wise CpG-sites (black) and between a-DMR CpG-sites (red) in the sample of 172 related individuals (solid line) and a subset of 96 unrelated individuals (dotted line).
(PDF)

Table S1 List of 490 a-DMRs.
(XLS)

Table S2 Descriptive statistics of the age-related phenotypes.
(XLS)

Table S3 List of 19 a-DMRs associated with proportion of lymphocytes.
(XLS)

Table S4 Overlap across genotype-methylation (cis-meQTLs), methylation-phenotype (ap-DMRs), and genotype-phenotype (GWAS) association results.
(XLS)

Table S5 JASPAR motif search results in the set of a-DMR genes. Results are shown at P = 0.05 threshold.
(XLS)

Table S6 Gene Ontology term enrichment results in the set of a-DMR genes. GO term enrichment in a-DMR genes was assessed relative to the background set of 14,344 genes that map nearest to the 26,690 probes tested. Results are shown at P = 1e-6 for biological processes and molecular functions.
(XLS)
