# Supplementary figures and images for: Epigenome-Wide Scans Identify Differentially Methylated Regions for Age and Age-Related Phenotypes in a Healthy Ageing Population
Source: PLoS Genet. 2012 Apr 19;8(4):e1002629. doi: 10.1371/journal.pgen.1002629 (PMC3330116; doi:10.1371/journal.pgen.1002629)

**A. Autosomes**

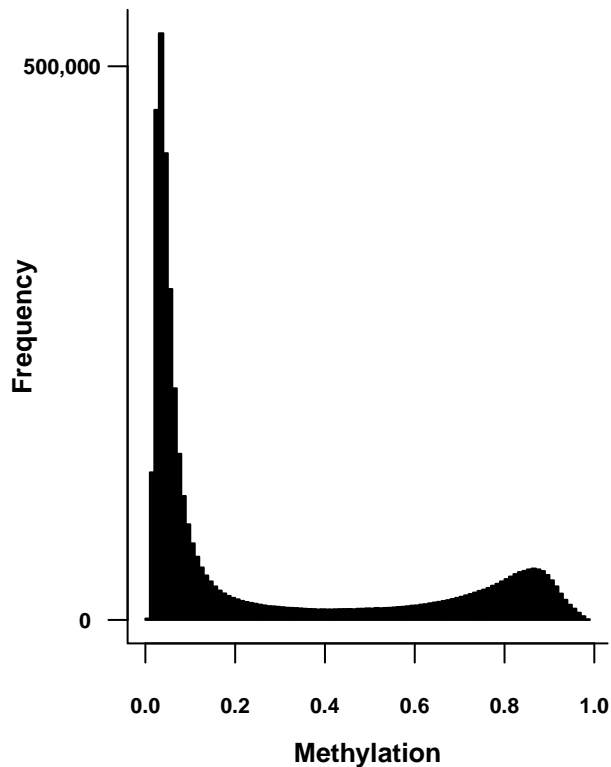

**B. X-chromosome**

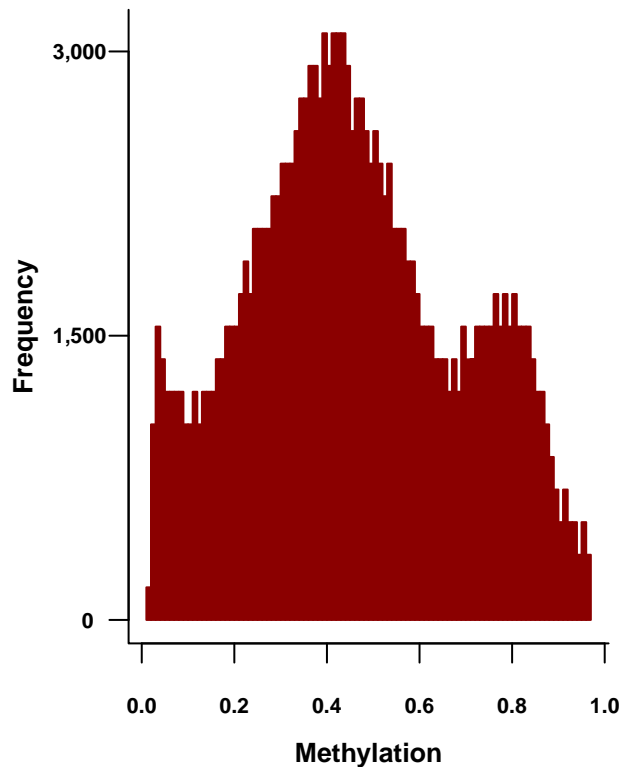

Supplement: Figure S1 — Summary characteristics of DNA methylation patterns in 172 female twins. Distribution of methylation scores (beta) in (A) autosomal and (B) X-chromosomal probes in all individuals. (PDF) [file pgen.1002629.s001.pdf]

**Batch 1: 29 twins pairs**

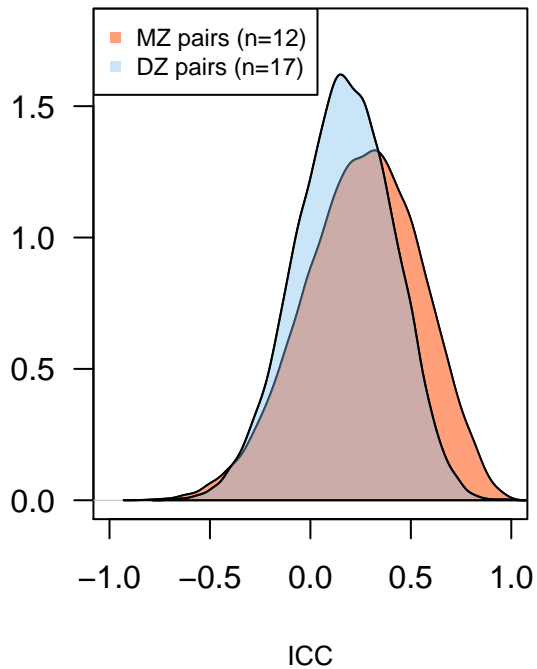

**Batch 2: 23 twins pairs**

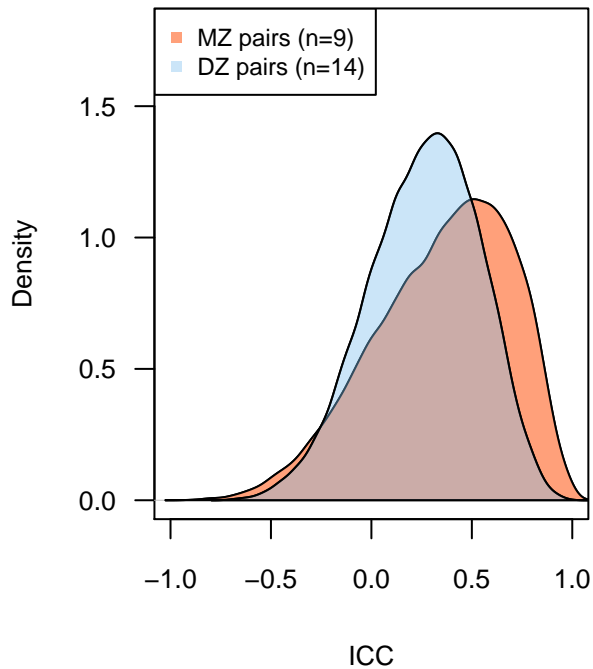

Supplement: Figure S2 — Distribution of intra-class correlation coefficients (ICC) in twins. Density plots of ICC in MZ twins (red) and DZ twins (blue) for two batches of methylation data (batch 1 consists of 93 twins (left) and batch 2 consists of 79 twins (right)). The mean MZ-ICCs and DZ-ICCs were estimated as 0.257 and 0.168 in batch 1 (MZ-ICC vs DZ-ICC P<2×10^−16^), and as 0.3557 and 0.261 in batch 2 (MZ-ICC vs DZ-ICC P<2×10^−16^). The corresponding methylation probe heritabilities were calculated as 2(ICC_MZ - ICC_DZ) and the genome-wide estimates were 0.176 (95%CI:0.168–0.185) and 0.188 (95%CI:0.180–0.196) for the data in batch 1 (left) and batch 2 (right), respectively. (PDF) [file pgen.1002629.s002.pdf]

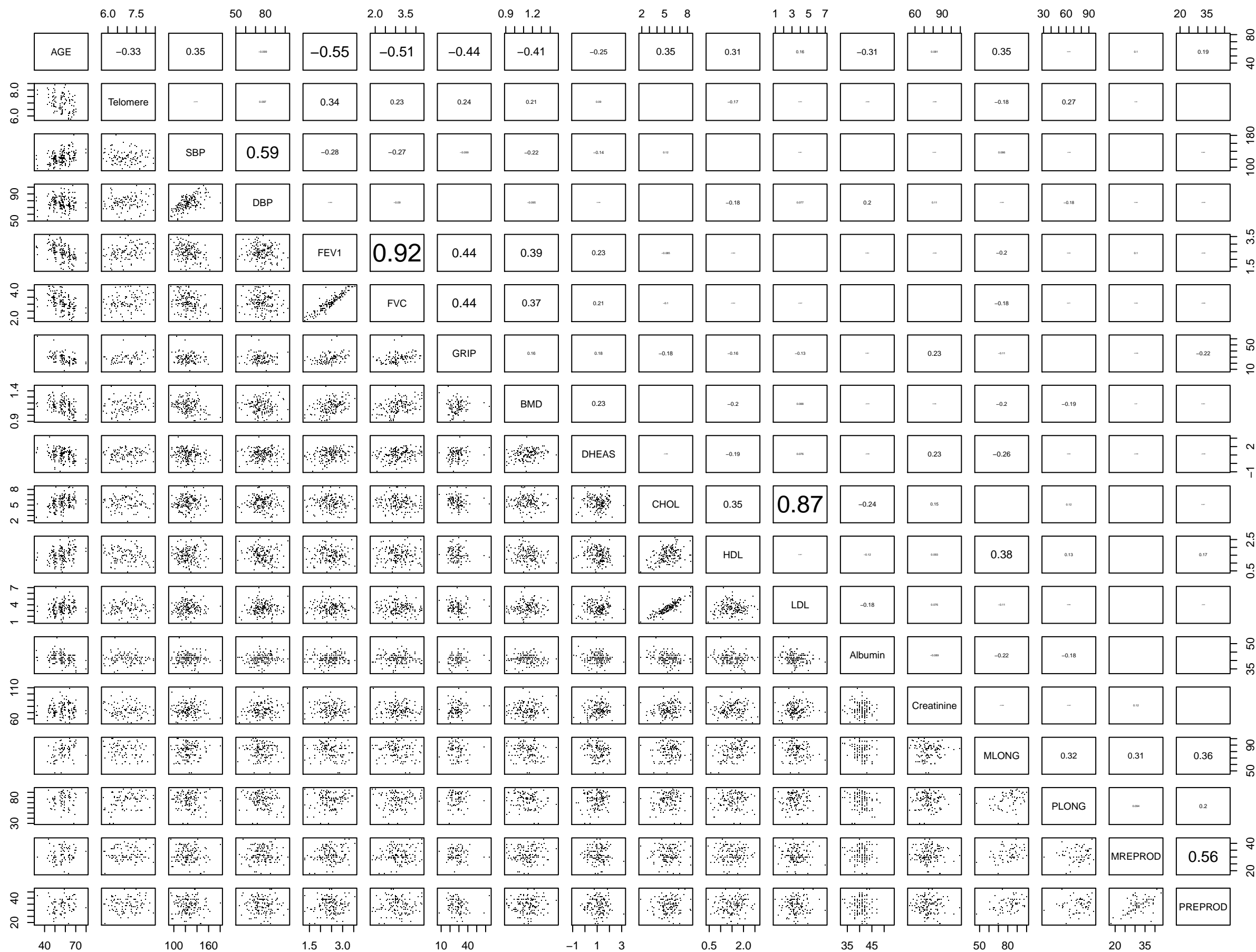

Supplement: Figure S3 — Correlation across age-related phenotypes. Below diagonal plots represent each pair of phenotypes and the corresponding rank correlation coefficient is shown above the diagonal. (PDF) [file pgen.1002629.s003.pdf]

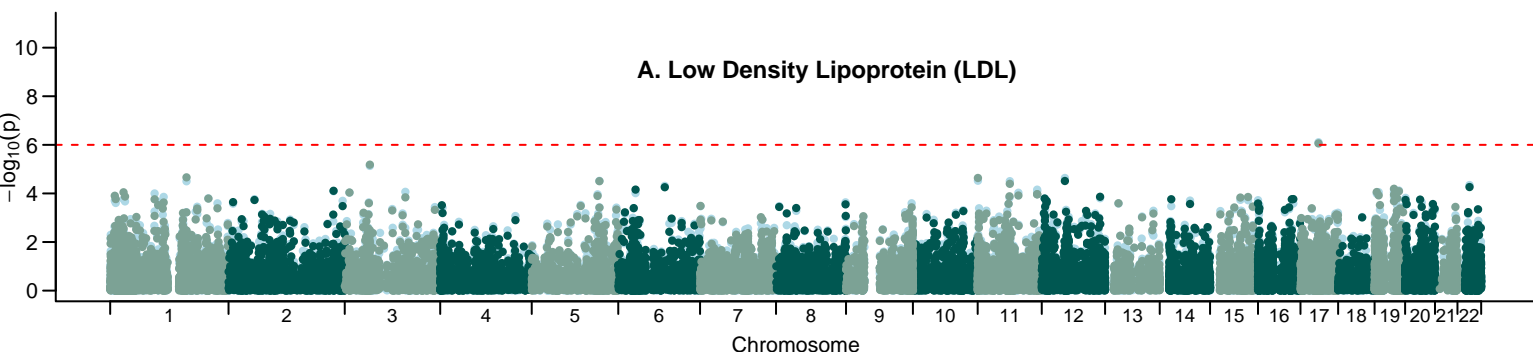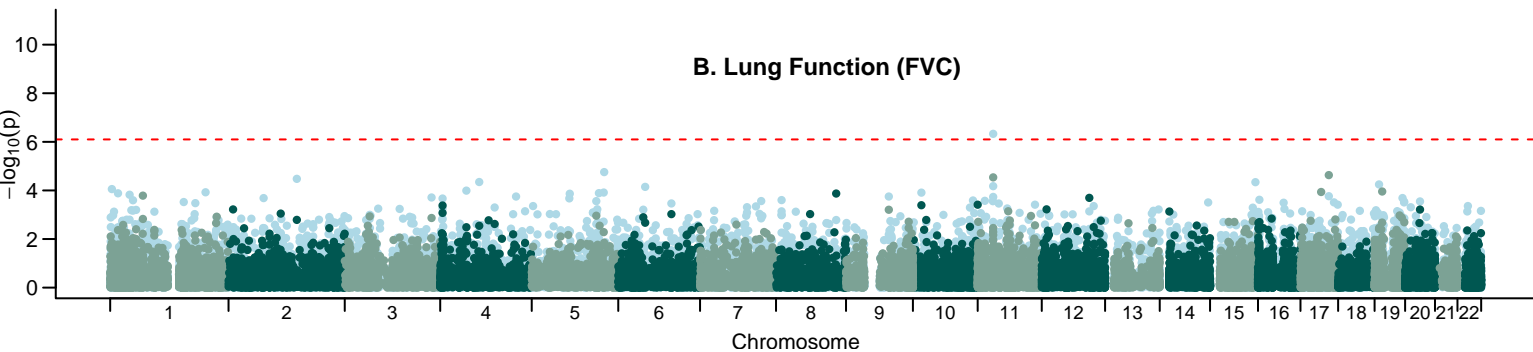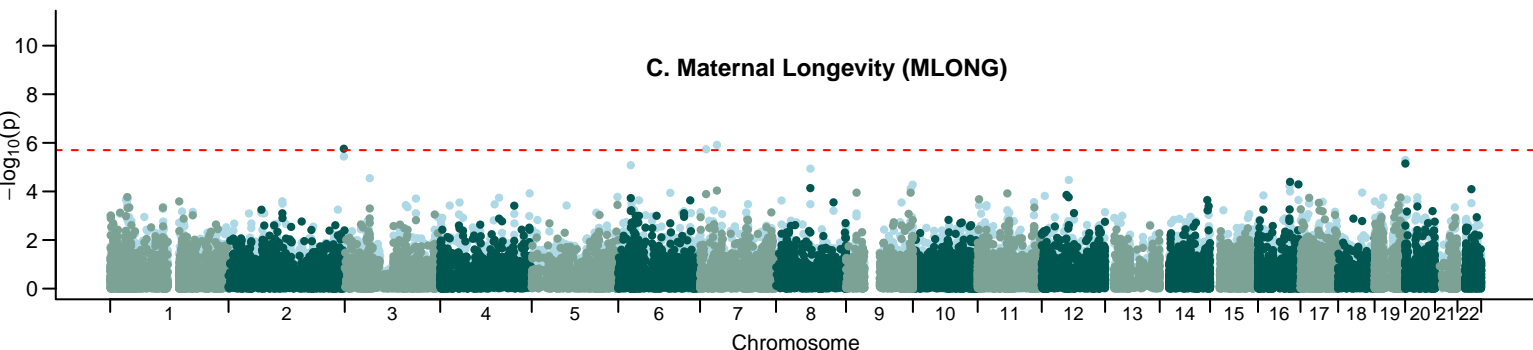

Supplement: Figure S4 — EWAS results for age-related phenotypes. FDR 5% ap-DMRs were obtained for (A) LDL, (B) lung function (FVC), and (C) maternal longevity (MLONG) with (green) and without (blue) age-correction. Red dashed lines correspond to age-corrected (A) and non-age-corrected (B,C) analysis FDR 5% levels. (PDF) [file pgen.1002629.s004.pdf]

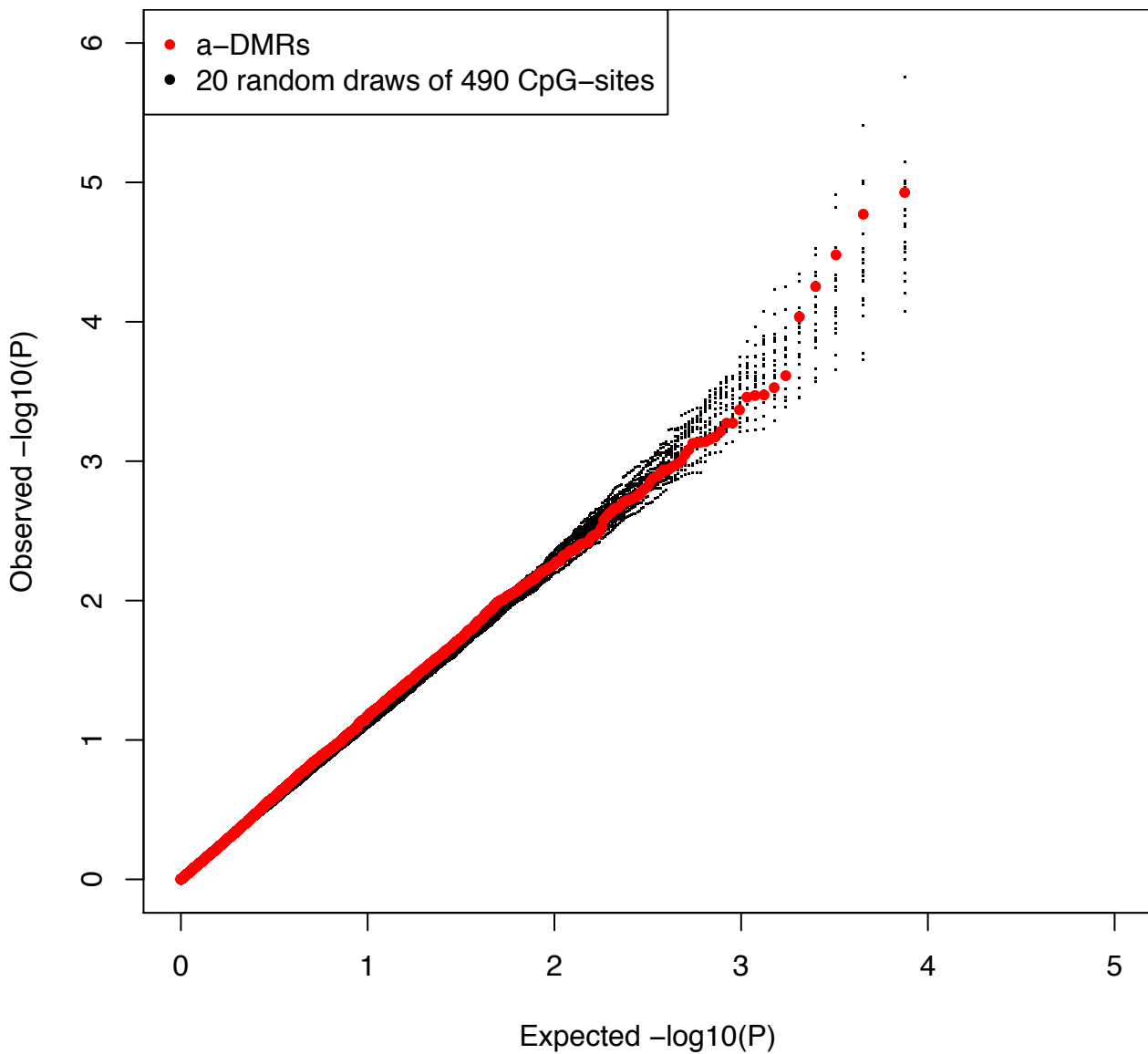

Supplement: Figure S5 — Lack of enrichment of age-related phenotype DMR association in the set of age DMRs. (PDF) [file pgen.1002629.s005.pdf]

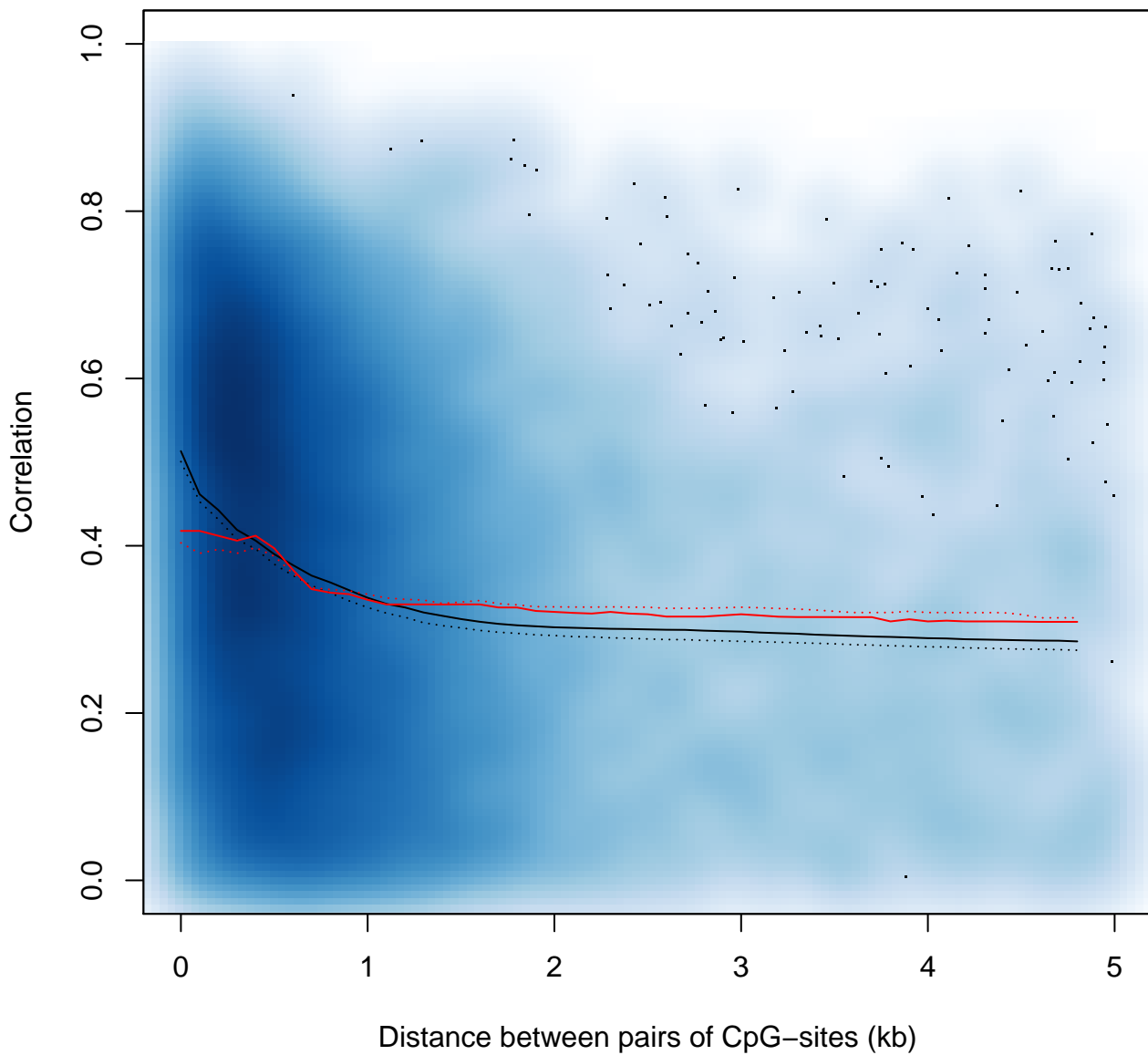

Supplement: Figure S6 — Evidence for co-methylation. Spearman correlation in methylation levels between all pair-wise CpG-sites (black) and between a-DMR CpG-sites (red) in the sample of 172 related individuals (solid line) and a subset of 96 unrelated individuals (dotted line). (PDF) [file pgen.1002629.s006.pdf]
